# Coexistence of Guillain-Barré Syndrome and Subacute Combined Degeneration of the Spinal Cord Due to Autoimmune Gastritis: A Case Report and Literature Review

**DOI:** 10.7759/cureus.63084

**Published:** 2024-06-25

**Authors:** Huiyao Xiang, Moushan Cai

**Affiliations:** 1 Department of Neurology, The First College of Clinical Medical Science, China Three Gorges University, Yichang, CHN

**Keywords:** methylcobalamin, immunoglobulin, autoimmune gastritis, subacute combined degeneration, guillain-barré syndrome

## Abstract

Guillain-Barré syndrome (GBS) and autoimmune gastritis (AIG) are both autoimmune diseases (ADs) that have a low prevalence in China. Both conditions involve the immune system mistakenly attacking the body's own tissues. GBS primarily affects the peripheral nervous system, leading to muscle weakness and paralysis, while AIG targets the stomach lining, causing inflammation and reduced absorption of vital nutrients. Subacute combined degeneration (SCD) of the spinal cord is the most common neurological manifestation of vitamin B12 deficiency. As of yet, there have been no reported cases of patients with GBS and complications of AIG including SCD. We report a case of a 54-year-old male patient who had been experiencing progressive numbness and weakness in his extremities, burning and tingling sensations, a cotton-stepping sensation, and difficulty walking for three weeks. He was admitted to the hospital and underwent an extensive medical workup. Magnetic resonance imaging (MRI) of the cervical spine cord showed abnormal spinal cord signal intensity consistent with typical manifestations of vitamin B12 deficiency. Gastric endoscopy revealed local atrophy of the gastric corpus, and gastric tissue biopsy indicated atrophic gastritis with intestinal metaplasia, consistent with a diagnosis of AIG. Lumbar puncture of cerebrospinal fluid (CSF) results showed albumincytological dissociation, further confirming the diagnosis of GBS. He was treated with intravenous immunoglobulin and methylcobalamin therapy for these conditions and showed significant clinical improvement upon discharge.

## Introduction

Guillain-Barré syndrome (GBS) is a rare autoimmune disorder (AD) that primarily impacts the peripheral nerves. It usually occurs after an infection or immune system activation, leading to an abnormal autoimmune response against the peripheral nerves and spinal roots. Molecular mimicry between microbial and nerve antigens is a significant factor in the development of the disorder. *Campylobacter jejuni* is the most common microorganism associated with GBS. The disease usually peaks at around two to four weeks and is often accompanied by albumincytological dissociation of the cerebrospinal fluid (CSF), mostly monophasic and self-limiting, and is effectively treated with intravenous immunoglobulin (IVIG) or plasma exchange (PE) [1,2]. Autoimmune gastritis (AIG) is an organ-specific, immune-mediated disease characterized by the destruction of parietal cells, loss of intrinsic factors, and decreased gastric acid excretion, also known as type A gastritis. These changes lead to malabsorption of iron, vitamin B12, and other micronutrients [3]. Subacute combined degeneration (SCD) of the spinal cord is the most common neurologic manifestation of vitamin B12 deficiency. It is usually secondary to AIG but can also occur in malnutritional syndromes, such as chronic alcoholism, strict vegetarianism, gastrectomy, and abuse of nitrous oxide [4,5]. Neurologic symptoms of SCD are mainly characterized by damage to the posterior and lateral cords of the spinal cord and peripheral nerves. They may also be accompanied by optic nerve damage, and in a few cases, symptoms of central nervous system involvement may occur, and treatment with high-dose vitamin B12 supplementation is effective [6]. In this paper, we report a case of GBS combined with AIG and SCD.

## Case presentation

A 54-year-old male patient presented with progressive numbness and weakness in the limbs for three weeks. Three weeks ago, he experienced numbness and weakness in both lower limbs, without any apparent cause. This condition was accompanied by an unsteady gait and a feeling of stepping on cotton. However, he could not take care of himself and did not receive any examination or treatment. Two weeks ago, he developed a fever with a peak temperature of 38.4°C, accompanied by nasal congestion, runny nose, cough, and sputum. At the same time, the numbness and weakness of both lower limbs worsened, and he needed help from his family to walk. One day ago, he began to experience numbness and weakness in both upper limbs, unable to hold objects, and difficulty in lifting. However, he did not experience slurred speech, dysphagia, choking on water, dyspnea, and dysuria. There was no other relevant past medical history.

During the neurologic examination, he showed grade 4 proximal muscle strength, grade 3 distal muscle strength, and grade 0 grip strength in both upper limbs. Meanwhile, he showed grade 5- proximal muscle strength and grade 3 distal muscle strength in both lower limbs. There was no muscle tenderness on palpation. There was decreased acupuncture sensation below the elbow joint in both upper limbs and below the ankle joint in both lower limbs, tendon reflexes of the limbs were absent, and Romberg's test was positive. The pathological reflex in cranial nerves was negative.

Laboratory examination revealed the following abnormalities: decreased red blood cell count (3.11 × 10^12^/L, normal range 3.8-5.1 × 10^12^/L), decreased hemoglobin (Hb) (84 g/L, normal range 115-150g/L), increased mean corpuscular volume (MCV) (114.8 fL, normal range 82-100 fL), increased mean corpuscular hemoglobin (38.9 pg, normal range 27-34 pg), decreased vitamin B12 levels (<50 pg/mL, normal range 187-883 pg/mL), increased homocysteine (HCY) levels (105.69 umol/L, normal range <15 umol/L), and positive anti-intrinsic factor antibodies and anti-parietal cell antibodies. The lumbar puncture CSF examination showed normal white blood cell count (4×10^6^/L, normal range 0-8×10^6^/L) and increased protein levels (189.80 mg/dL, normal range 15-45 mg/dL). Furthermore, the C13 urea breath test, blood potassium, blood copper, muscle enzyme profile, thyroid function, tumor markers, autoantibody profile, immunoglobulin antibodies, anti-MOG antibodies, AQP4 antibodies, oligoclonal bands, and pathogenicity tests (including *Campylobacter jejuni, *Epstein-Barr virus, cytomegalovirus, Zika virus, varicella-zoster virus, human immunodeficiency virus antigenic antibody, and syphilis antibody) were all normal.

**Figure 1:**
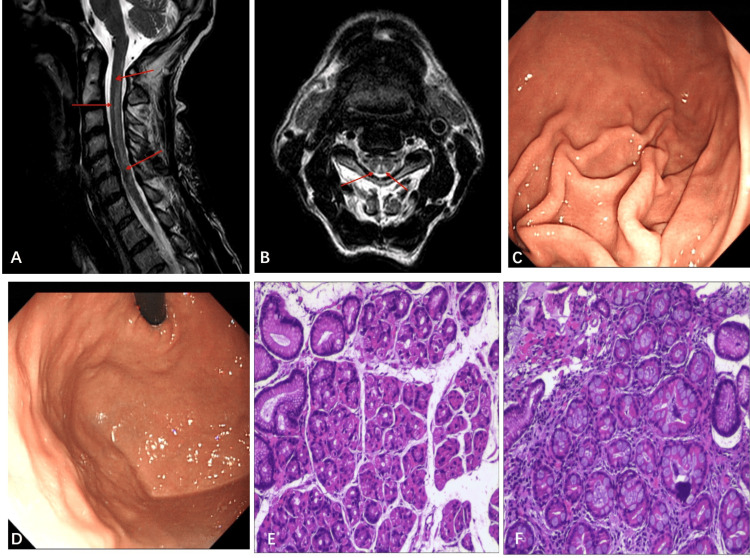
Magnetic resonance image (MRI) findings in the spinal cord. (A) abnormal longitudinally extensive T2-weighted hyperintensities involving the posterior columns (the red arrow), (B) inverted V or “rabbit ears” sign on cervical spinal (the red arrow). The gastroscope shows the atrophic gastritis in corpus (C) and fundus (D). Histopathology of the gastric mucosa shows atrophy and intestinal metaplasia in the mucosa (E and F).

Brain MRI showed nonspecific cerebral white matter lesions, and the optic nerve showed no abnormality. Cervical spinal cord MRI showed an abnormally longitudinally extensive T2-weighted hyperintensities involving the posterior columns with an inverted V or “rabbit ears” sign (Figure 1A-1B), which was a typical manifestation of SCD. Nerve conduction velocity (NCV) revealed peripheral neurogenic damage to the nerves of both upper and lower extremities bilaterally, involving both motor and sensory functions, with prolonged F-wave latency. He did not have any digestive symptoms and had not taken any gastric medication, including proton pump inhibitors. Gastroscopy showed thin mucosa in the fundus and corpus, with a marked visible vascular pattern and fold atrophy (Figure 1C-1D). Biopsies of the fundus and corpus revealed atrophy and intestinal metaplasia (Figure 1E-1F), which are typical signs of AIG.

Our final diagnosis is as follows: 1) GBS, 2) AIG, 3) subacute combined degeneration of the spinal cord, 4) macrocytic anemia, and 5) vitamin B12 deficiency. For treatment, we used immunoglobulin 0.4g/(kg·d) for five days, as well as methylcobalamin 1000 μg/d for intravenous push, and adjuvant treatment including vitamin B1, folic acid, and iron. By the fourth day of treatment, the patient's symptoms, such as limb weakness and numbness, gradually improved, and he was able to walk independently with grade 5- proximal muscle strength, grade 4 distal muscle strength of the limbs, and grade 2 grip strength of both hands. After three weeks of treatment, the patient was discharged with muscle strength graded as 5, hand grip strength as 4, and some residual numbness below the wrist and ankle joints bilaterally. Blood tests showed normal hemoglobin levels. Because it would be difficult for the patient to continue receiving intravenous methylcobalamin after discharge, we recommended taking oral methylcobalamin 1500 ug/d for life. A follow-up call six months post-discharge indicated that the patient had almost completely recovered from limb numbness and weakness symptoms.

## Discussion

GBS is a type of acute inflammatory polyradiculoneuropathy that is the most common cause of acute flaccid paralysis worldwide. It is a group of autoimmune peripheral neuropathies that can be triggered by infections, vaccinations, and surgeries. It can happen at any age and season and usually manifests as an acute monophasic course that is self-limiting [7]. The main clinical features of GBS include flaccid limb muscle weakness that typically starts from the lower limbs and progresses to the upper limbs, often peaking within two to four weeks. It may also be accompanied by distal sensory deficits of the extremities, pain or soreness in the lower limbs, nerve trunk pressure, and pulling pain. Diagnosis of the disorder may be supported by albumin cytological dissociation of the CSF and NCV peripheral nerve damage [8]. In some cases, CSF oligoclonal bands and positive anti-ganglioside antibodies may be observed. Our case involves a patient who experienced an acute onset of illness with a respiratory infection and presented with symmetrical and delayed paralysis. The neurophysiological examination was suggestive of peripheral nerve damage in the extremities, which was staged as acute inflammatory demyelinating polyneuropathy (AIDP). According to the diagnostic criteria established by the European Academy of Neurology in 2023 [9], our case meets the diagnosis of GBS.

The typical symptoms of GBS are usually easy to diagnose, but ignoring atypical changes may lead to a missed diagnosis. Neurophysiological tests and CSF analysis are essential to support the diagnosis, even though they may initially show normal results [10]. Patients should be carefully monitored for any signs of worsening, especially for medullary palsy, respiratory weakness, and autonomic dysfunction. There are prognostic scales available to help predict progression and determine the best course of treatment [11,12]. Currently, IVIG and PE are the recognized treatments for speeding up recovery, but some patients may still not survive or be unable to walk independently even with these treatments [8]. New treatments such as antibody therapy, complement pathway interventions, and inhibition of inflammatory cells and factors are currently being actively researched [2].

Conditions that need to be distinguished from GBS are acute myelitis and spinal infarction. Acute myelitis presents with a sudden onset of motor deficits, sensory deficits, and bladder and rectal sphincter dysfunction below the affected area within hours or days. Typical MRI findings include thickening of the spinal cord at the site of the lesion and multiple lamellar or speckled lesions within the affected segments, showing T1-weighted low signal, T2-weighted high signal, uneven signal intensity, and visible fusion. However, some cases may not show these abnormalities. Spinal infarction usually has an acute onset, but in some instances, it can have a slower onset within a few days. Most cases are associated with underlying atherosclerotic diseases, such as hypertension and diabetes mellitus. Symptoms include sudden onset of painful numbness, often manifesting as radicular pain at the level corresponding to the spinal cord segments, along with weakness and loss of sensation. Characteristic changes include dissociative sensory deficits below the level of the lesion, such as loss of pain and temperature sensation and the presence of deep sensation. Spinal MRI may show T2-weighted high signal and spinal cord enlargement, while diffusion-weighted imaging (DWI) may reveal marked cytotoxic edema and diffusion restriction, which help differentiate acute spinal cord ischemia from other spinal cord lesions. 

AIG stands for autoimmune chemotactic atrophic gastritis, which is a chronic inflammatory disease of the stomach caused by an autoimmune response that attacks the parietal cells and intrinsic factors. This results in a lack of gastric acid, increased levels of gastrin, and decreased secretion of intrinsic factor [13]. Patients with AIG experience reduced secretion of essential substances such as gastric acid and intrinsic factors, which can lead to digestive dysfunction. Currently, there are no globally standardized criteria for diagnosing AIG, so we refer to the Japanese diagnostic criteria [14]. It involves two conditions: positive specific autoantibodies, such as anti-parietal cell antibodies and/or anti-intrinsic factor antibodies, and severe mucosal atrophy from the body of the stomach to the fundus of the stomach visible during gastroscopy. If both conditions are met, the case can be confirmed. In addition, the serologic examination of gastric mucosal function, such as serum gastrin and pepsinogen levels, can be useful in diagnosing AIG. In this case, although there were no gastrointestinal symptoms, such as abdominal distension and dyspepsia, the presence of positive antibodies to mural cells and endogastric factors, and pathological changes in the gastric mucosa suggest the presence of AIG, accompanied by systemic lesions such as reduced vitamin B12 levels and megaloblastic anemia.

The pathogenesis of AIG mainly involves CD4+ T-lymphocytes targeting the H+/K+-ATPase of the parietal cells, which then stimulates the plasma cells to produce autoantibodies. These autoantibodies include anti-parietal cell antibodies and intrinsic factor antibodies [15]. The former plays a key role in parietal cell destruction and glandular atrophy [16], while the latter is an intrinsic mechanism leading to vitamin B12 deficiency and pernicious anemia [17]. Although the presence of autoantibodies is important for diagnosing AIG, their absence does not completely exclude the disease. The titer of anti-parietal cell antibodies may gradually decrease during the different stages of development of the disease, and patients with advanced disease may even show negative anti-parietal cell antibodies due to the absence of parietal cells [18]. Furthermore, studies indicate that the absence of anti-parietal cell antibodies is more common in elderly patients with AIG [19]. Meanwhile, AIG typically remains in a "quiescent phase" during the early stages, leading to delayed diagnosis due to the lack of specific symptoms. Therefore, clinical suspicion is crucial for diagnosis.

SCD is a rare neurodegenerative disease that affects the posterior and lateral cords of the spinal cord and peripheral nerves. It is characterized by the demyelination of the lateral and dorsal columns of the spinal cord. The main symptoms of SCD are weakness, paresthesia, unsteady gait, and positive Romberg, which are often early manifestations of the disease. CSF examination is usually normal. MRI of the cervical and upper thoracic spinal cord often shows striated and speckled lesions with T1-weighted low signal and T2-weighted high signal. On NCV, both sensory and motor nerves are impaired, mainly with demyelinating lesions and some axonal damage.

SCD is caused by impaired intake, absorption, combination, transport, or metabolism of vitamin B12. AIG can lead to severe malabsorption of vitamin B12. Other causes of vitamin B12 deficiency include inadequate intake due to a prolonged vegetarian diet, gastrectomy, ileostomy, impaired absorption due to prolonged treatment with proton pump inhibitors, alcoholism with atrophic gastritis, and nitrous oxide inhalation [20]. If a patient is diagnosed with SCD, further investigations should be performed to identify the underlying cause and give effective treatment [21]. In this case, the patient came in with complaints of numbness, weakness in the limbs, a feeling of cotton-thread sensation, and unsteady gait. The laboratory tests indicated a decrease in vitamin B12 and an increase in Hcy. Furthermore, an MRI of the cervical spine cord revealed an inverted V or "rabbit ear" sign. Based on these findings, the diagnosis of SCD was confirmed.

The patient fulfilled the diagnostic criteria of GBS, AIG, and SCD. The presence of these diseases has been reported in the past, but to the best of our knowledge, this is the first overlapping case of GBS, AIG, and SCD. SCD is usually a neurologic condition caused by vitamin B12 deficiency, and after screening the patient for risk factors associated with vitamin B12 deficiency, it was ultimately determined to be due to AIG.

It is important to note that AIG, as an AD, often coexists with other ADs. Autoimmune thyroid disease is the most common comorbidity in patients with AIG [22]. Other ADs that are known to coexist with AIG include type 1 diabetes mellitus, rheumatoid arthritis, primary dry syndrome, systemic lupus erythematosus, autoimmune hepatitis, ankylosing spondylitis, celiac disease, inflammatory bowel disease, vitiligo, chronic lipitis, myasthenia gravis, Addison's disease, and others [23,24]. This indicates that comorbidity of AIG with other ADs is not uncommon. In this context, multiple autoimmunity is defined as the presence of more than one AD in a single patient. If three or more ADs are present in a patient at the same time, it is referred to as multiple autoimmune syndrome. Studies have demonstrated that 34.4% of patients with ADs have multiple ADs concurrently, with autoimmune thyroid disease and dry syndrome being the most common among these patients [25].

GBS is a rare AD, and there are no reports of AIG combined with GBS. Although AIG is associated with other ADs, the mechanisms of comorbidity remain to be elucidated. Molecular mimicry mechanisms can provide valuable insights into the pathogenesis of ADs by elucidating key molecular interactions [26]. A study showed that some lipopolysaccharide components of *Helicobacter pylori *(*H. pylori*) may have similar structures to certain glycolipids in neural tissues. When the body is infected with *H. pylori*, the immune system produces antibodies against *H. pylori*. Some of these antibodies may misrecognize and attack the nerve tissues due to molecular mimicry, leading to neuroinflammation and demyelination, thus causing GBS [27]. In addition, it has been shown that *H. pylori *can express antigens that are structurally similar to the gastric parietal cell H+/K+-ATPase (an autoantigen), which leads to cross-reactivity of gastric T-cells to these two antigens. This cross-reactivity activates and amplifies the gastric auto-reactive T cells against H+/K+-ATPase, which in turn attacks the gastric parietal cells, leading to parietal cell destruction and gastric atrophy. Damage to gastric parietal cells also exposes new autoantigens, which, through epitope spreading mechanisms, activate T cells directed against other gastric autoantigens, leading to more severe gastric inflammation and ultimately to AIG or gastric atrophy [28]. Taken together, these findings emphasize the complex interplay of *H. pylori *infection, molecular mimicry, and cross-reactivity in the pathogenesis of various ADs, such as GBS and AIG.

## Conclusions

This is an interesting case of acute GBS with concomitant SCD and AD. The case highlights the complex interplay of AD and nutritional deficiencies in the development of neurological disorders. It underscores the importance of a thorough evaluation and treatment for such cases. The rare combination of GBS and SCD due to AIG presents a unique and complex medical scenario. For patients with this type of condition, it is important to work closely with a team of professionals, including neurologists, gastroenterologists, and other specialists, to develop a comprehensive treatment plan. Furthermore, it is crucial to raise awareness among healthcare professionals about the rare neurological manifestations associated with ADs to enable early detection and timely intervention. This case serves as a reminder to remain vigilant in patients presenting with atypical neurologic symptoms and to thoroughly investigate and address all possible underlying causes.

## References

[1] D'Elios MM, Appelmelk BJ, Amedei A, Bergman MP, Del Prete G (2004). Gastric autoimmunity: the role of Helicobacter pylori and molecular mimicry. Trends Mol Med.

[2] Rojas-Villarraga A, Amaya-Amaya J, Rodriguez-Rodriguez A, Mantilla RD, Anaya JM (2012). Introducing polyautoimmunity: secondary autoimmune diseases no longer exist. Autoimmune Dis.

[3] Killen JP, Brenninger VL (2013). Vitamin B12 deficiency. N Engl J Med.

[4] Oldstone MB (2014). Molecular mimicry: its evolution from concept to mechanism as a cause of autoimmune diseases. Monoclon Antib Immunodiagn Immunother.

[5] Kalkan Ç, Soykan I (2016). Polyautoimmunity in autoimmune gastritis. Eur J Intern Med.

[6] Willison HJ, Jacobs BC, van Doorn PA (2016). Guillain-Barré syndrome. Lancet.

[7] Cellini M, Santaguida MG, Virili C, Capriello S, Brusca N, Gargano L, Centanni M (2017). Hashimoto's thyroiditis and autoimmune gastritis. Front Endocrinol (Lausanne).

[8] Minalyan A, Benhammou JN, Artashesyan A, Lewis MS, Pisegna JR (2017). Autoimmune atrophic gastritis: current perspectives. Clin Exp Gastroenterol.

[9] Cao J, Su ZY, Xu SB, Liu CC (2018). Subacute combined degeneration: a retrospective study of 68 cases with short-term follow-up. Eur Neurol.

[10] Rodriguez-Castro KI, Franceschi M, Miraglia C (2018). Autoimmune diseases in autoimmune atrophic gastritis. Acta Biomed.

[11] Zhang N, Li RH, Ma L, Li N, Shan PY, Wang XB, Liu AF (2019). Subacute combined degeneration, pernicious anemia and gastric neuroendocrine tumor occured simultaneously caused by autoimmune gastritis. Front Neurosci.

[12] Conti L, Lenti MV, Di Sabatino A (2020). Seronegative autoimmune atrophic gastritis is more common in elderly patients. Dig Liver Dis.

[13] Dardiotis E, Sokratous M, Tsouris Z (2020). Association between Helicobacter pylori infection and Guillain-Barré Syndrome: a meta-analysis. Eur J Clin Invest.

[14] Lenti MV, Rugge M, Lahner E (2020). Autoimmune gastritis. Nat Rev Dis Primers.

[15] Kumar M, Kalita J, Kant Misra U, Dhar N (2021). Prediction models for mechanical ventilation and outcome in Guillain-Barré syndrome. J Clin Neurosci.

[16] Livzan MA, Gaus OV, Mozgovoi SI, Bordin DS (2021). Chronic autoimmune gastritis: modern diagnostic principles. Diagnostics (Basel).

[17] Shahrizaila N, Lehmann HC, Kuwabara S (2021). Guillain-Barré syndrome. Lancet.

[18] Linazi G, Abudureyimu S, Zhang J (2022). Clinical features of different stage subacute combined degeneration of the spinal cord. Medicine (Baltimore).

[19] Nishizawa T, Watanabe H, Yoshida S (2022). Decreased anti-parietal cell antibody titer in the advanced phase of autoimmune gastritis. Scand J Gastroenterol.

[20] Yoon JY, Klein JP (2022). Subacute combined degeneration from nitrous oxide use. N Engl J Med.

[21] Allakky A (2023). Exploring the association of Helicobacter pylori with anti-intrinsic factor and anti-parietal cell antibodies in pernicious anemia: a systematic review. Cureus.

[22] Kamada T, Watanabe H, Furuta T (2023). Diagnostic criteria and endoscopic and histological findings of autoimmune gastritis in Japan. J Gastroenterol.

[23] Song Y, Zheng X, Fang Y, Liu S, Liu K, Zhu J, Wu X (2023). Current status of Guillain-Barré syndrome (GBS) in China: a 10-year comprehensive overview. Rev Neurosci.

[24] Yao J, Zhou R, Liu Y, Liu Y, Cao Q, Lu Z (2023). Predicting of mechanical ventilation and outcomes by using models and biomarker in Guillain-Barré syndrome. Neurol Ther.

[25] Yao J, Zhou R, Liu Y, Lu Z (2023). Progress in Guillain-Barré syndrome immunotherapy-a narrative review of new strategies in recent years. Hum Vaccin Immunother.

[26] Yu YF, Tong KK, Shangguan XL (2023). Research status and hotspots of autoimmune gastritis: a bibliometric analysis. World J Gastroenterol.

[27] Zaki HA, Iftikhar H, Najam M (2023). Plasma exchange (PE) versus intravenous immunoglobulin (IVIG) for the treatment of Guillain-Barré syndrome (GBS) in patients with severe symptoms: a systematic review and meta-analysis. eNeurologicalSci.

[28] van Doorn PA, Van den Bergh PY, Hadden RD (2024). Response to letter on European Academy of Neurology/Peripheral Nerve Society guideline on diagnosis and treatment of Guillain-Barré syndrome. Eur J Neurol.

